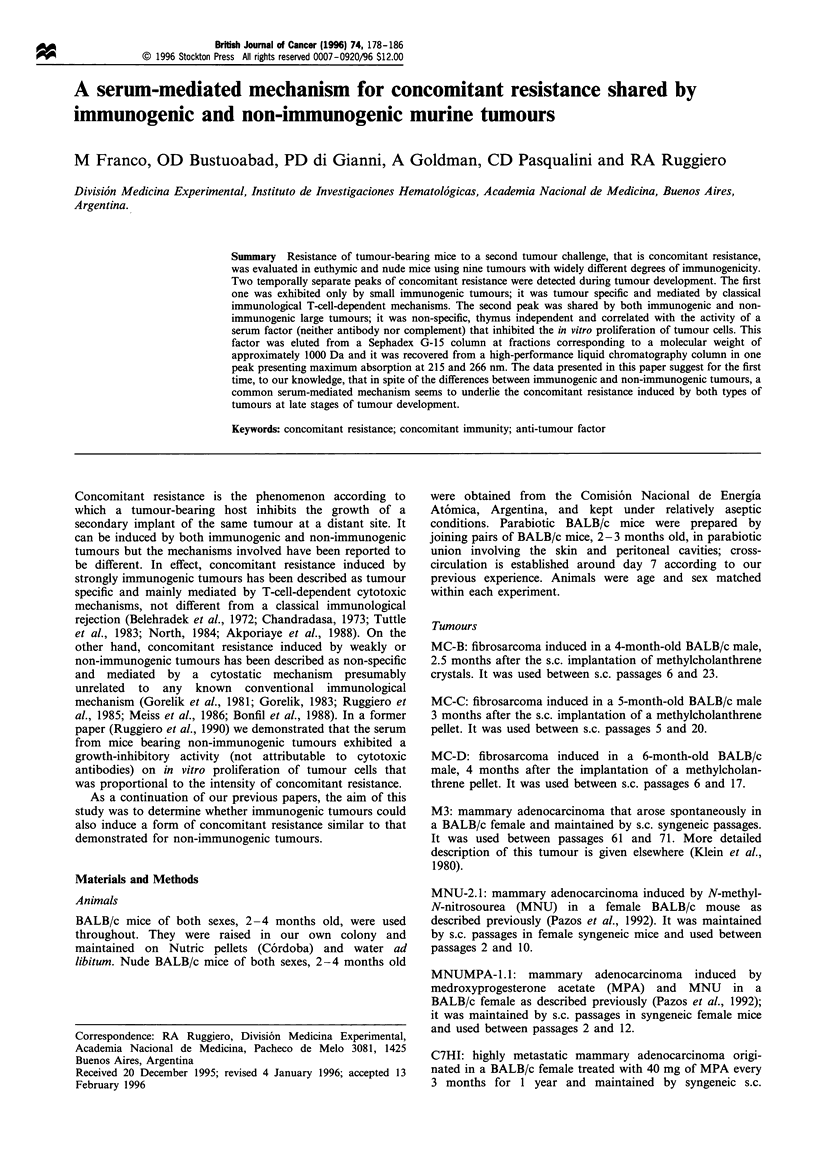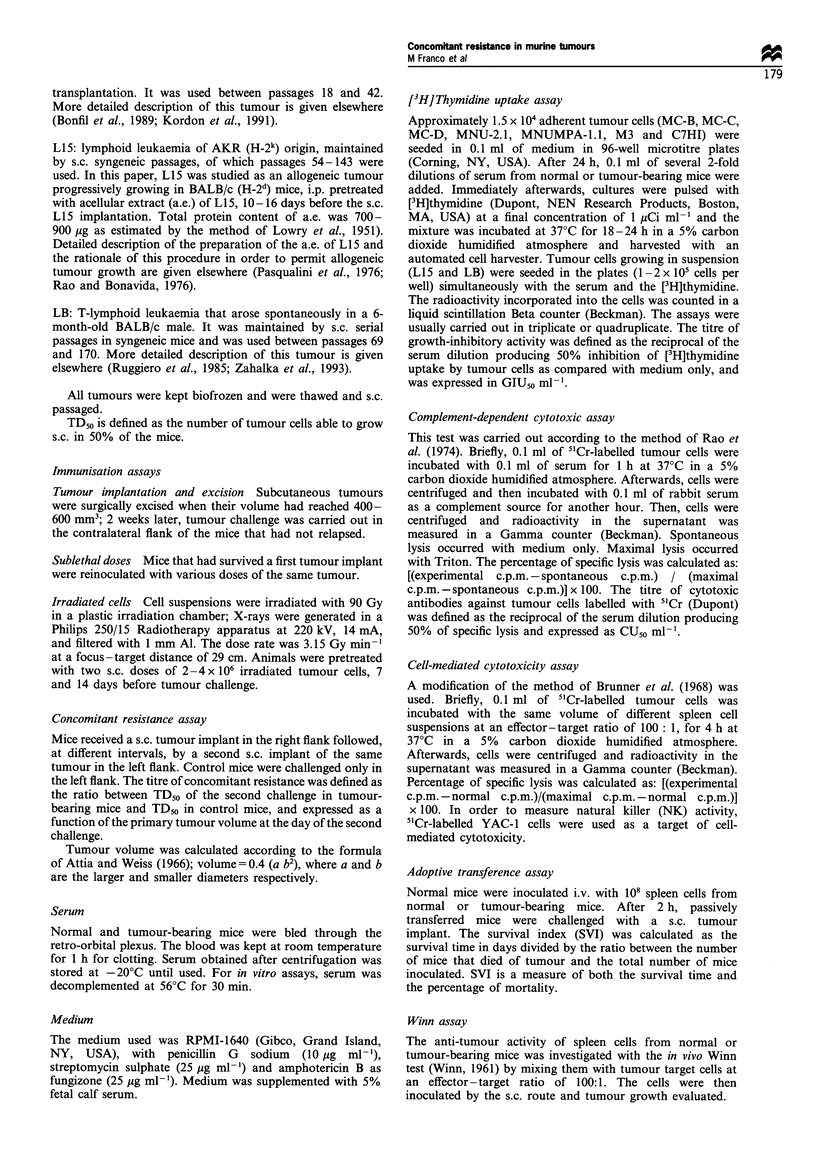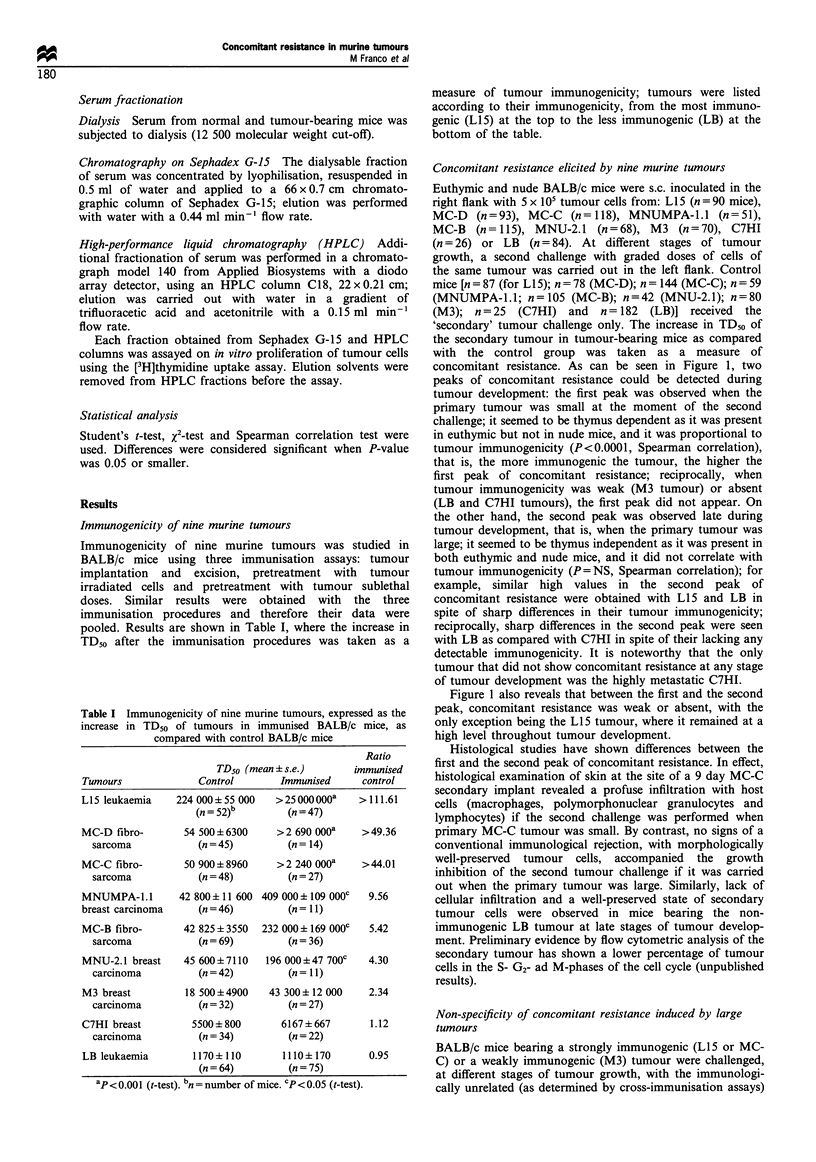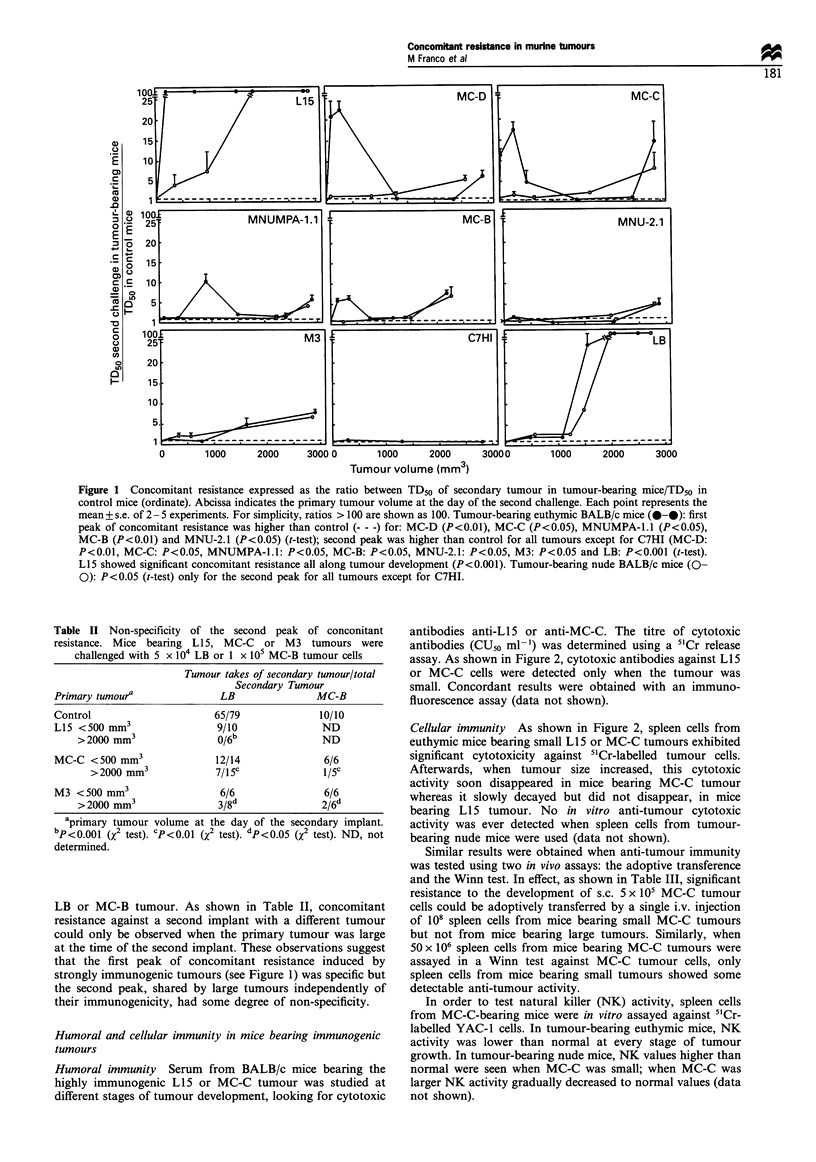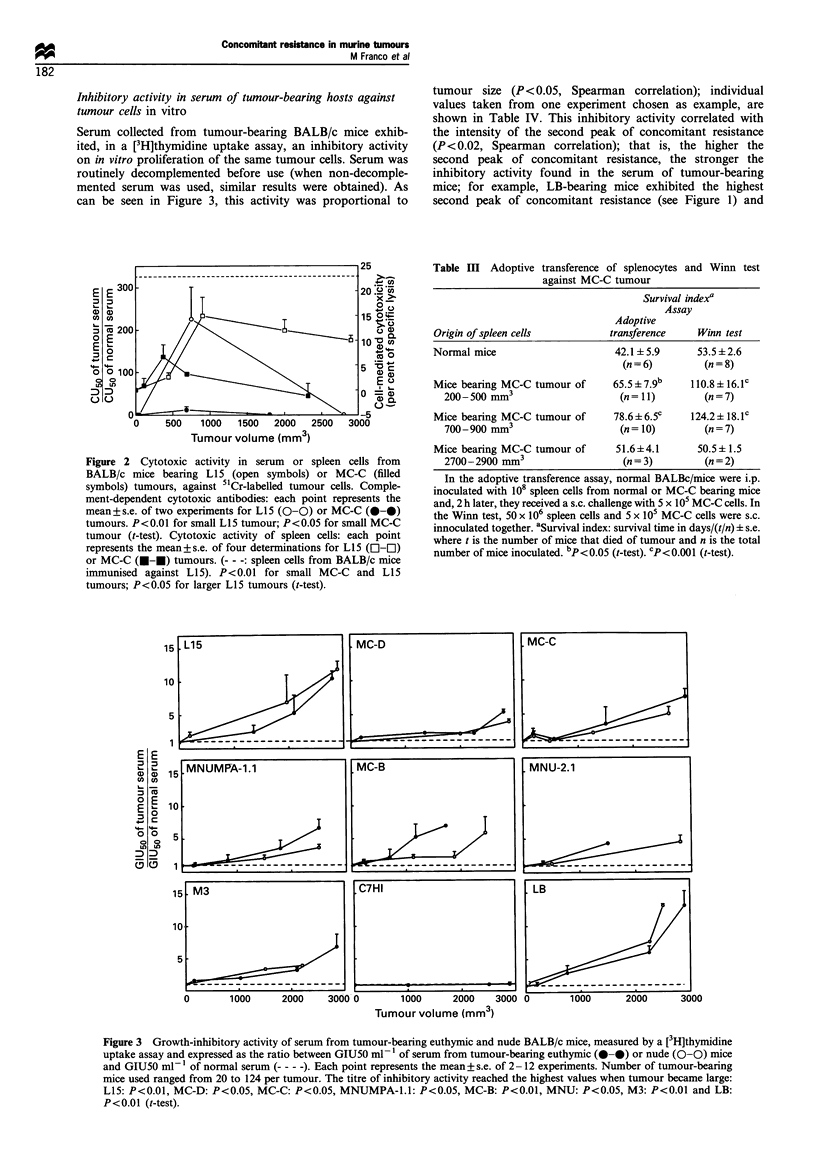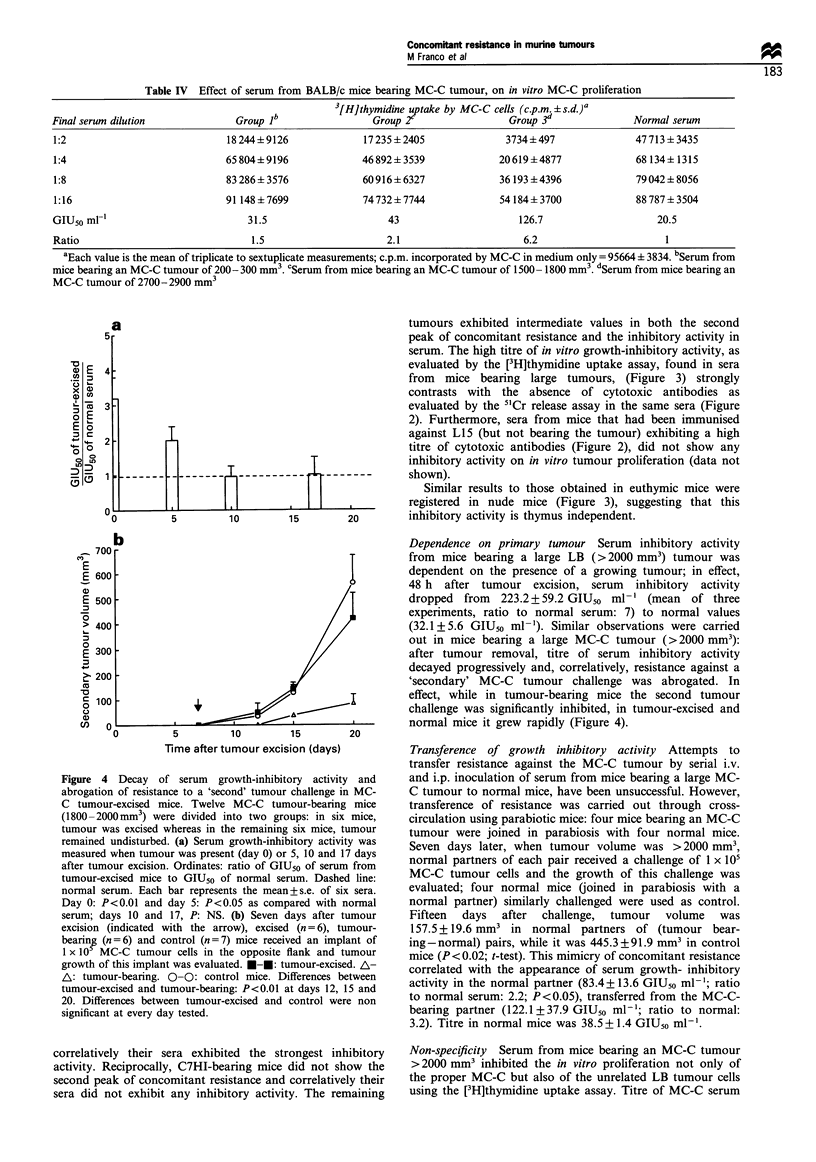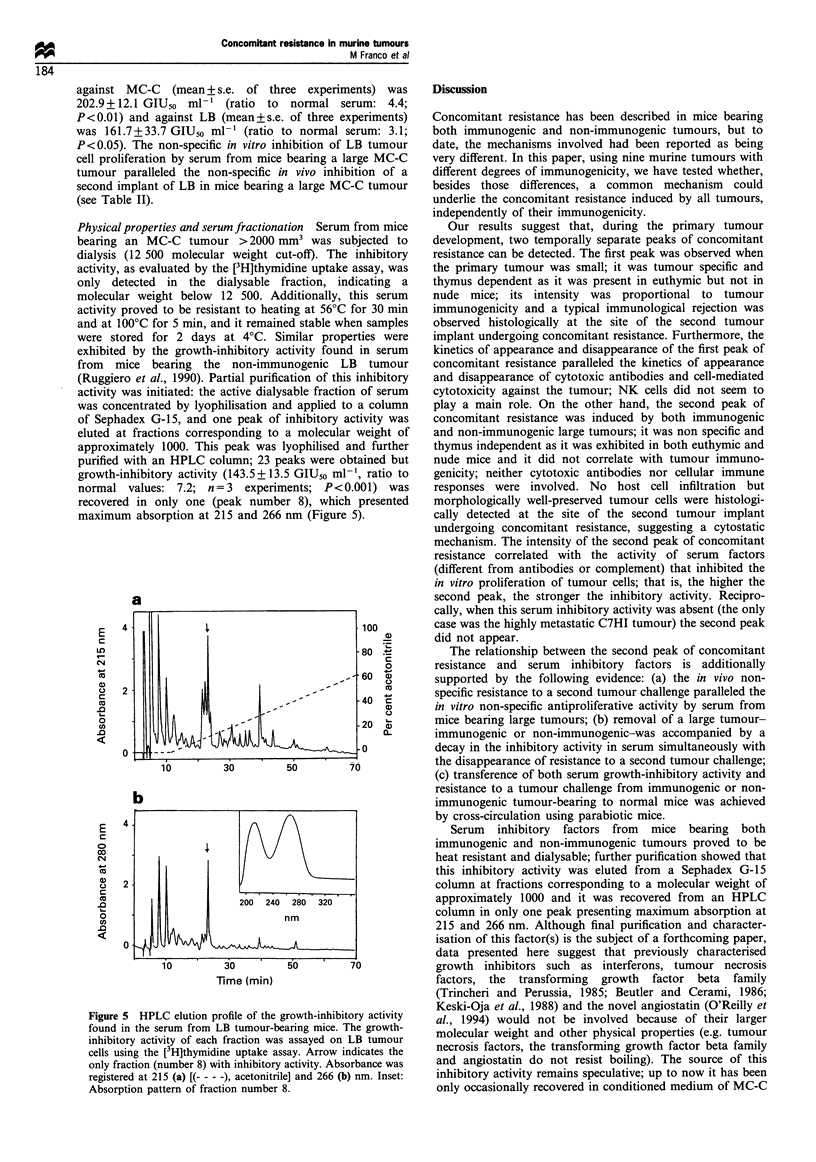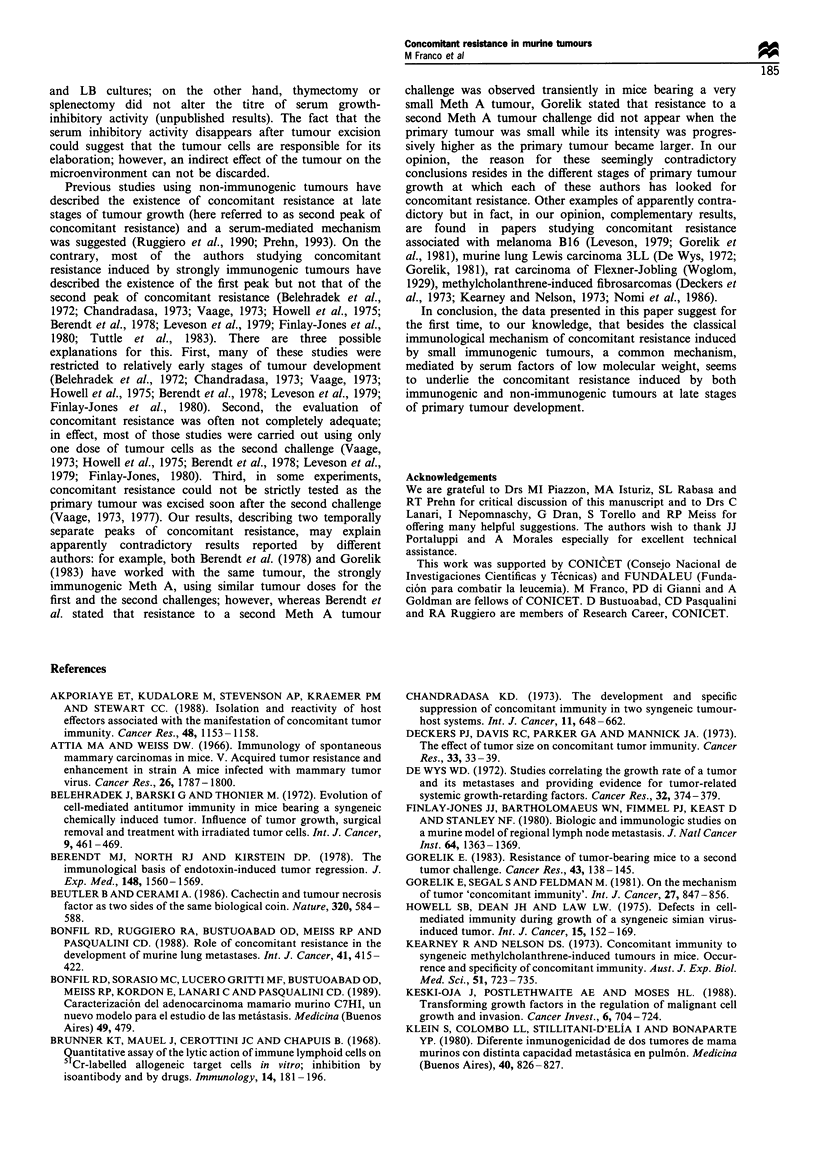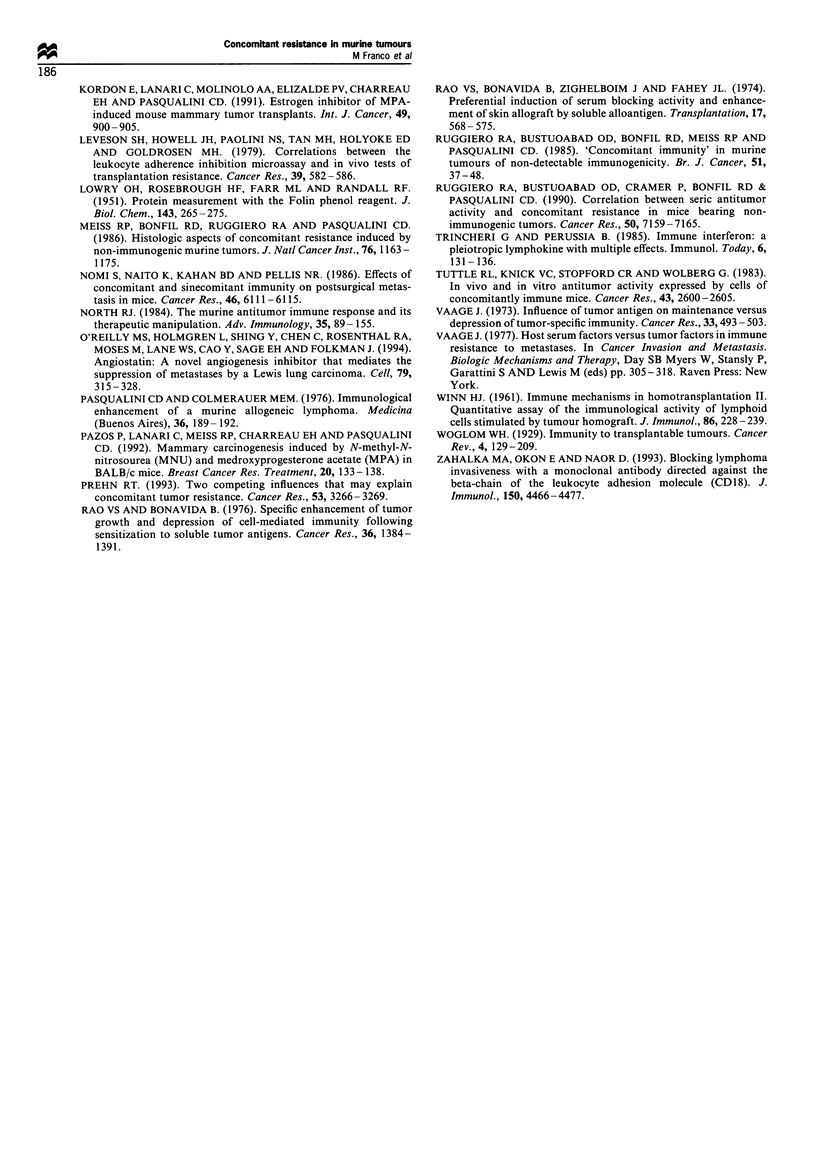# A serum-mediated mechanism for concomitant resistance shared by immunogenic and non-immunogenic murine tumours.

**DOI:** 10.1038/bjc.1996.335

**Published:** 1996-07

**Authors:** M. Franco, O. D. Bustuoabad, P. D. di Gianni, A. Goldman, C. D. Pasqualini, R. A. Ruggiero

**Affiliations:** División Medicina Experimental, Academia Nacional de Medicina, Buenos Aires, Argentina.

## Abstract

Resistance of tumour-bearing mice to a second tumour challenge, that is concomitant resistance, was evaluated in euthymic and nude mice using nine tumours with widely different degrees of immunogenicity. Two temporally separate peaks of concomitant resistance were detected during tumour development. The first one was exhibited only by small immunogenic tumours; it was tumour specific and mediated by classical immunological T-cell-dependent mechanisms. The second peak was shared by both immunogenic and non-immunogenic large tumours; it was non-specific, thymus independent and correlated with the activity of a serum factor (neither antibody nor complement) that inhibited the in vitro proliferation of tumour cells. This factor was eluted from a Sephadex G-15 column at fractions corresponding to a molecular weight of approximately 1000 Da and it was recovered from a high-performance liquid chromatography column in one peak presenting maximum absorption at 215 and 266 nm. The data presented in this paper suggest for the first time, to our knowledge, that in spite of the differences between immunogenic and non-immunogenic tumours, a common serum-mediated mechanism seems to underlie the concomitant resistance induced by both types of tumours at late stages of tumour development.


British Journal of Cancer (1996) 74, 178-186
© 1996 Stockton Press All rights reserved 0007-0920/96 $12.00

### A serum-mediated mechanism for concomitant resistance shared by immunogenic and non-immunogenic murine tumours

M Franco, OD Bustuoabad, PD di Gianni, A Goldman, CD Pasqualini and RA Ruggiero

*División Medicina Experimental, Instituto de Investigaciones Hematológicas, Academia Nacional de Medicina, Buenos Aires, Argentina.*

**Summary** Resistance of tumour-bearing mice to a second tumour challenge, that is concomitant resistance, was evaluated in euthymic and nude mice using nine tumours with widely different degrees of immunogenicity. Two temporally separate peaks of concomitant resistance were detected during tumour development. The first one was exhibited only by small immunogenic tumours; it was tumour specific and mediated by classical immunological T-cell-dependent mechanisms. The second peak was shared by both immunogenic and non-immunogenic large tumours; it was non-specific, thymus independent and correlated with the activity of a serum factor (neither antibody nor complement) that inhibited the *in vitro* proliferation of tumour cells. This factor was eluted from a Sephadex G-15 column at fractions corresponding to a molecular weight of approximately 1000 Da and it was recovered from a high-performance liquid chromatography column in one peak presenting maximum absorption at 215 and 266 nm. The data presented in this paper suggest for the first time, to our knowledge, that in spite of the differences between immunogenic and non-immunogenic tumours, a common serum-mediated mechanism seems to underlie the concomitant resistance induced by both types of tumours at late stages of tumour development.

**Keywords:** concomitant resistance; concomitant immunity; anti-tumour factor

Concomitant resistance is the phenomenon according to which a tumour-bearing host inhibits the growth of a secondary implant of the same tumour at a distant site. It can be induced by both immunogenic and non-immunogenic tumours but the mechanisms involved have been reported to be different. In effect, concomitant resistance induced by strongly immunogenic tumours has been described as tumour specific and mainly mediated by T-cell-dependent cytotoxic mechanisms, not different from a classical immunological rejection (Belehradek *et al.*, 1972; Chandradasa, 1973; Tuttle *et al.*, 1983; North, 1984; Akporiaye *et al.*, 1988). On the other hand, concomitant resistance induced by weakly or non-immunogenic tumours has been described as non-specific and mediated by a cytostatic mechanism presumably unrelated to any known conventional immunological mechanism (Gorelik *et al.*, 1981; Gorelik, 1983; Ruggiero *et al.*, 1985; Meiss *et al.*, 1986; Bonfil *et al.*, 1988). In a former paper (Ruggiero *et al.*, 1990) we demonstrated that the serum from mice bearing non-immunogenic tumours exhibited a growth-inhibitory activity (not attributable to cytotoxic antibodies) on *in vitro* proliferation of tumour cells that was proportional to the intensity of concomitant resistance.

As a continuation of our previous papers, the aim of this study was to determine whether immunogenic tumours could also induce a form of concomitant resistance similar to that demonstrated for non-immunogenic tumours.

### Materials and Methods

### *Animals*

BALB/c mice of both sexes, 2-4 months old, were used throughout. They were raised in our own colony and maintained on Nutric pellets (Córdoba) and water *ad libitum*. Nude BALB/c mice of both sexes, 2-4 months old were obtained from the Comisión Nacional de Energía Atómica, Argentina, and kept under relatively aseptic conditions. Parabiotic BALB/c mice were prepared by joining pairs of BALB/c mice, 2-3 months old, in parabiotic union involving the skin and peritoneal cavities; cross-circulation is established around day 7 according to our previous experience. Animals were age and sex matched within each experiment.

### *Tumours*

MC-B: fibrosarcoma induced in a 4-month-old BALB/c male, 2.5 months after the s.c. implantation of methylcholanthrene crystals. It was used between s.c. passages 6 and 23.

MC-C: fibrosarcoma induced in a 5-month-old BALB/c male 3 months after the s.c. implantation of a methylcholanthrene pellet. It was used between s.c. passages 5 and 20.

MC-D: fibrosarcoma induced in a 6-month-old BALB/c male, 4 months after the implantation of a methylcholanthrene pellet. It was used between s.c. passages 6 and 17.

M3: mammary adenocarcinoma that arose spontaneously in a BALB/c female and maintained by s.c. syngeneic passages. It was used between passages 61 and 71. More detailed description of this tumour is given elsewhere (Klein *et al.*, 1980).

MNU-2.1: mammary adenocarcinoma induced by *N*-methyl-*N*-nitrosourea (MNU) in a female BALB/c mouse as described previously (Pazos *et al.*, 1992). It was maintained by s.c. passages in female syngeneic mice and used between passages 2 and 10.

MNUMPA-1.1: mammary adenocarcinoma induced by medroxyprogesterone acetate (MPA) and MNU in a BALB/c female as described previously (Pazos *et al.*, 1992); it was maintained by s.c. passages in syngeneic female mice and used between passages 2 and 12.

C7HI: highly metastatic mammary adenocarcinoma originated in a BALB/c female treated with 40 mg of MPA every 3 months for 1 year and maintained by syngeneic s.c.

Correspondence: RA Ruggiero, División Medicina Experimental, Academia Nacional de Medicina, Pacheco de Melo 3081, 1425 Buenos Aires, Argentina
Received 20 December 1995; revised 4 January 1996; accepted 13 February 1996

transplantation. It was used between passages 18 and 42. More detailed description of this tumour is given elsewhere (Bonfil *et al.*, 1989; Kordon *et al.*, 1991).

L15: lymphoid leukaemia of AKR ($H\text{-}2^k$) origin, maintained by s.c. syngeneic passages, of which passages 54–143 were used. In this paper, L15 was studied as an allogeneic tumour progressively growing in BALB/c ($H\text{-}2^d$) mice, i.p. pretreated with acellular extract (a.e.) of L15, 10–16 days before the s.c. L15 implantation. Total protein content of a.e. was 700–900 μg as estimated by the method of Lowry *et al.*, 1951). Detailed description of the preparation of the a.e. of L15 and the rationale of this procedure in order to permit allogeneic tumour growth are given elsewhere (Pasqualini *et al.*, 1976; Rao and Bonavida, 1976).

LB: T-lymphoid leukaemia that arose spontaneously in a 6-month-old BALB/c male. It was maintained by s.c. serial passages in syngeneic mice and was used between passages 69 and 170. More detailed description of this tumour is given elsewhere (Ruggiero *et al.*, 1985; Zahalka *et al.*, 1993).

All tumours were kept biofrozen and were thawed and s.c. passaged.

$TD_{50}$ is defined as the number of tumour cells able to grow s.c. in 50% of the mice.

### *Immunisation assays*

*Tumour implantation and excision* Subcutaneous tumours were surgically excised when their volume had reached 400–600 $mm^3$; 2 weeks later, tumour challenge was carried out in the contralateral flank of the mice that had not relapsed.

*Sublethal doses* Mice that had survived a first tumour implant were reinoculated with various doses of the same tumour.

*Irradiated cells* Cell suspensions were irradiated with 90 Gy in a plastic irradiation chamber; X-rays were generated in a Philips 250/15 Radiotherapy apparatus at 220 kV, 14 mA, and filtered with 1 mm Al. The dose rate was 3.15 Gy $min^{-1}$ at a focus–target distance of 29 cm. Animals were pretreated with two s.c. doses of $2-4 \times 10^6$ irradiated tumour cells, 7 and 14 days before tumour challenge.

### *Concomitant resistance assay*

Mice received a s.c. tumour implant in the right flank followed, at different intervals, by a second s.c. implant of the same tumour in the left flank. Control mice were challenged only in the left flank. The titre of concomitant resistance was defined as the ratio between $TD_{50}$ of the second challenge in tumour-bearing mice and $TD_{50}$ in control mice, and expressed as a function of the primary tumour volume at the day of the second challenge.

Tumour volume was calculated according to the formula of Attia and Weiss (1966); volume = 0.4 ($a\ b^2$), where $a$ and $b$ are the larger and smaller diameters respectively.

### *Serum*

Normal and tumour-bearing mice were bled through the retro-orbital plexus. The blood was kept at room temperature for 1 h for clotting. Serum obtained after centrifugation was stored at −20°C until used. For *in vitro* assays, serum was decomplemented at 56°C for 30 min.

### *Medium*

The medium used was RPMI-1640 (Gibco, Grand Island, NY, USA), with penicillin G sodium (10 μg $ml^{-1}$), streptomycin sulphate (25 μg $ml^{-1}$) and amphotericin B as fungizone (25 μg $ml^{-1}$). Medium was supplemented with 5% fetal calf serum.

### *[$^3H$]Thymidine uptake assay*

Approximately $1.5 \times 10^4$ adherent tumour cells (MC-B, MC-C, MC-D, MNU-2.1, MNUMPA-1.1, M3 and C7HI) were seeded in 0.1 ml of medium in 96-well microtitre plates (Corning, NY, USA). After 24 h, 0.1 ml of several 2-fold dilutions of serum from normal or tumour-bearing mice were added. Immediately afterwards, cultures were pulsed with [$^3H$]thymidine (Dupont, NEN Research Products, Boston, MA, USA) at a final concentration of 1 μCi $ml^{-1}$ and the mixture was incubated at 37°C for 18–24 h in a 5% carbon dioxide humidified atmosphere and harvested with an automated cell harvester. Tumour cells growing in suspension (L15 and LB) were seeded in the plates ($1-2 \times 10^5$ cells per well) simultaneously with the serum and the [$^3H$]thymidine. The radioactivity incorporated into the cells was counted in a liquid scintillation Beta counter (Beckman). The assays were usually carried out in triplicate or quadruplicate. The titre of growth-inhibitory activity was defined as the reciprocal of the serum dilution producing 50% inhibition of [$^3H$]thymidine uptake by tumour cells as compared with medium only, and was expressed in $GIU_{50}$ $ml^{-1}$.

### *Complement-dependent cytotoxic assay*

This test was carried out according to the method of Rao *et al.* (1974). Briefly, 0.1 ml of $^{51}Cr$-labelled tumour cells were incubated with 0.1 ml of serum for 1 h at 37°C in a 5% carbon dioxide humidified atmosphere. Afterwards, cells were centrifuged and then incubated with 0.1 ml of rabbit serum as a complement source for another hour. Then, cells were centrifuged and radioactivity in the supernatant was measured in a Gamma counter (Beckman). Spontaneous lysis occurred with medium only. Maximal lysis occurred with Triton. The percentage of specific lysis was calculated as: [(experimental c.p.m. − spontaneous c.p.m.) / (maximal c.p.m. − spontaneous c.p.m.)] × 100. The titre of cytotoxic antibodies against tumour cells labelled with $^{51}Cr$ (Dupont) was defined as the reciprocal of the serum dilution producing 50% of specific lysis and expressed as $CU_{50}$ $ml^{-1}$.

### *Cell-mediated cytotoxicity assay*

A modification of the method of Brunner *et al.* (1968) was used. Briefly, 0.1 ml of $^{51}Cr$-labelled tumour cells was incubated with the same volume of different spleen cell suspensions at an effector–target ratio of 100 : 1, for 4 h at 37°C in a 5% carbon dioxide humidified atmosphere. Afterwards, cells were centrifuged and radioactivity in the supernatant was measured in a Gamma counter (Beckman). Percentage of specific lysis was calculated as: [(experimental c.p.m. − normal c.p.m.)/(maximal c.p.m. − normal c.p.m.)] × 100. In order to measure natural killer (NK) activity, $^{51}Cr$-labelled YAC-1 cells were used as a target of cell-mediated cytotoxicity.

### *Adoptive transference assay*

Normal mice were inoculated i.v. with $10^8$ spleen cells from normal or tumour-bearing mice. After 2 h, passively transferred mice were challenged with a s.c. tumour implant. The survival index (SVI) was calculated as the survival time in days divided by the ratio between the number of mice that died of tumour and the total number of mice inoculated. SVI is a measure of both the survival time and the percentage of mortality.

### *Winn assay*

The anti-tumour activity of spleen cells from normal or tumour-bearing mice was investigated with the *in vivo* Winn test (Winn, 1961) by mixing them with tumour target cells at an effector–target ratio of 100:1. The cells were then inoculated by the s.c. route and tumour growth evaluated.

*Serum fractionation*

*Dialysis* Serum from normal and tumour-bearing mice was subjected to dialysis (12 500 molecular weight cut-off).

*Chromatography on Sephadex G-15* The dialysable fraction of serum was concentrated by lyophilisation, resuspended in 0.5 ml of water and applied to a $66 \times 0.7$ cm chromatographic column of Sephadex G-15; elution was performed with water with a 0.44 ml $min^{-1}$ flow rate.

*High-performance liquid chromatography (HPLC)* Additional fractionation of serum was performed in a chromatograph model 140 from Applied Biosystems with a diodo array detector, using an HPLC column C18, $22 \times 0.21$ cm; elution was carried out with water in a gradient of trifluoracetic acid and acetonitrile with a 0.15 ml $min^{-1}$ flow rate.

Each fraction obtained from Sephadex G-15 and HPLC columns was assayed on *in vitro* proliferation of tumour cells using the [$^3$H]thymidine uptake assay. Elution solvents were removed from HPLC fractions before the assay.

*Statistical analysis*

Student's *t*-test, $\chi^2$-test and Spearman correlation test were used. Differences were considered significant when *P*-value was 0.05 or smaller.

### Results

*Immunogenicity of nine murine tumours*

Immunogenicity of nine murine tumours was studied in BALB/c mice using three immunisation assays: tumour implantation and excision, pretreatment with tumour irradiated cells and pretreatment with tumour sublethal doses. Similar results were obtained with the three immunisation procedures and therefore their data were pooled. Results are shown in Table I, where the increase in $TD_{50}$ after the immunisation procedures was taken as a measure of tumour immunogenicity; tumours were listed according to their immunogenicity, from the most immunogenic (L15) at the top to the less immunogenic (LB) at the bottom of the table.

**Table I** Immunogenicity of nine murine tumours, expressed as the increase in $TD_{50}$ of tumours in immunised BALB/c mice, as compared with control BALB/c mice

| *Tumours* | *$TD_{50}$ (mean ± s.e.)* Control | Immunised | *Ratio immunised control* |
|---|---|---|---|
| L15 leukaemia | 224 000 ± 55 000 ($n = 52$)[b] | > 25 000 000[a] ($n = 47$) | > 111.61 |
| MC-D fibrosarcoma | 54 500 ± 6300 ($n = 45$) | > 2 690 000[a] ($n = 14$) | > 49.36 |
| MC-C fibrosarcoma | 50 900 ± 8960 ($n = 48$) | > 2 240 000[a] ($n = 27$) | > 44.01 |
| MNUMPA-1.1 breast carcinoma | 42 800 ± 11 600 ($n = 46$) | 409 000 ± 109 000[c] ($n = 11$) | 9.56 |
| MC-B fibrosarcoma | 42 825 ± 3550 ($n = 69$) | 232 000 ± 169 000[c] ($n = 36$) | 5.42 |
| MNU-2.1 breast carcinoma | 45 600 ± 7110 ($n = 42$) | 196 000 ± 47 700[c] ($n = 11$) | 4.30 |
| M3 breast carcinoma | 18 500 ± 4900 ($n = 32$) | 43 300 ± 12 000 ($n = 27$) | 2.34 |
| C7HI breast carcinoma | 5500 ± 800 ($n = 34$) | 6167 ± 667 ($n = 22$) | 1.12 |
| LB leukaemia | 1170 ± 110 ($n = 64$) | 1110 ± 170 ($n = 75$) | 0.95 |

[a]$P < 0.001$ (*t*-test). [b]$n$ = number of mice. [c]$P < 0.05$ (*t*-test).

*Concomitant resistance elicited by nine murine tumours*

Euthymic and nude BALB/c mice were s.c. inoculated in the right flank with $5 \times 10^5$ tumour cells from: L15 ($n = 90$ mice), MC-D ($n = 93$), MC-C ($n = 118$), MNUMPA-1.1 ($n = 51$), MC-B ($n = 115$), MNU-2.1 ($n = 68$), M3 ($n = 70$), C7HI ($n = 26$) or LB ($n = 84$). At different stages of tumour growth, a second challenge with graded doses of cells of the same tumour was carried out in the left flank. Control mice [$n = 87$ (for L15); $n = 78$ (MC-D); $n = 144$ (MC-C); $n = 59$ (MNUMPA-1.1; $n = 105$ (MC-B); $n = 42$ (MNU-2.1); $n = 80$ (M3); $n = 25$ (C7HI) and $n = 182$ (LB)] received the 'secondary' tumour challenge only. The increase in $TD_{50}$ of the secondary tumour in tumour-bearing mice as compared with the control group was taken as a measure of concomitant resistance. As can be seen in Figure 1, two peaks of concomitant resistance could be detected during tumour development: the first peak was observed when the primary tumour was small at the moment of the second challenge; it seemed to be thymus dependent as it was present in euthymic but not in nude mice, and it was proportional to tumour immunogenicity ($P < 0.0001$, Spearman correlation), that is, the more immunogenic the tumour, the higher the first peak of concomitant resistance; reciprocally, when tumour immunogenicity was weak (M3 tumour) or absent (LB and C7HI tumours), the first peak did not appear. On the other hand, the second peak was observed late during tumour development, that is, when the primary tumour was large; it seemed to be thymus independent as it was present in both euthymic and nude mice, and it did not correlate with tumour immunogenicity ($P$ = NS, Spearman correlation); for example, similar high values in the second peak of concomitant resistance were obtained with L15 and LB in spite of sharp differences in their tumour immunogenicity; reciprocally, sharp differences in the second peak were seen with LB as compared with C7HI in spite of their lacking any detectable immunogenicity. It is noteworthy that the only tumour that did not show concomitant resistance at any stage of tumour development was the highly metastatic C7HI.

Figure 1 also reveals that between the first and the second peak, concomitant resistance was weak or absent, with the only exception being the L15 tumour, where it remained at a high level throughout tumour development.

Histological studies have shown differences between the first and the second peak of concomitant resistance. In effect, histological examination of skin at the site of a 9 day MC-C secondary implant revealed a profuse infiltration with host cells (macrophages, polymorphonuclear granulocytes and lymphocytes) if the second challenge was performed when primary MC-C tumour was small. By contrast, no signs of a conventional immunological rejection, with morphologically well-preserved tumour cells, accompanied the growth inhibition of the second tumour challenge if it was carried out when the primary tumour was large. Similarly, lack of cellular infiltration and a well-preserved state of secondary tumour cells were observed in mice bearing the non-immunogenic LB tumour at late stages of tumour development. Preliminary evidence by flow cytometric analysis of the secondary tumour has shown a lower percentage of tumour cells in the S- $G_2$- ad M-phases of the cell cycle (unpublished results).

*Non-specificity of concomitant resistance induced by large tumours*

BALB/c mice bearing a strongly immunogenic (L15 or MC-C) or a weakly immunogenic (M3) tumour were challenged, at different stages of tumour growth, with the immunologically unrelated (as determined by cross-immunisation assays)

**Figure 1** Concomitant resistance expressed as the ratio between $TD_{50}$ of secondary tumour in tumour-bearing mice/$TD_{50}$ in control mice (ordinate). Abcissa indicates the primary tumour volume at the day of the second challenge. Each point represents the mean ± s.e. of 2–5 experiments. For simplicity, ratios >100 are shown as 100. Tumour-bearing euthymic BALB/c mice (●–●): first peak of concomitant resistance was higher than control (- - -) for: MC-D ($P<0.01$), MC-C ($P<0.05$), MNUMPA-1.1 ($P<0.05$), MC-B ($P<0.01$) and MNU-2.1 ($P<0.05$) (*t*-test); second peak was higher than control for all tumours except for C7HI (MC-D: $P<0.01$, MC-C: $P<0.05$, MNUMPA-1.1: $P<0.05$, MC-B: $P<0.05$, MNU-2.1: $P<0.05$, M3: $P<0.05$ and LB: $P<0.001$ (*t*-test). L15 showed significant concomitant resistance all along tumour development ($P<0.001$). Tumour-bearing nude BALB/c mice (○–○): $P<0.05$ (*t*-test) only for the second peak for all tumours except for C7HI.

**Table II** Non-specificity of the second peak of conconitant resistance. Mice bearing L15, MC-C or M3 tumours were challenged with $5 \times 10^4$ LB or $1 \times 10^5$ MC-B tumour cells

| | *Tumour takes of secondary tumour/total* | |
|---|---|---|
| | *Secondary Tumour* | |
| *Primary tumour*[a] | *LB* | *MC-B* |
| Control | 65/79 | 10/10 |
| L15 <500 mm$^3$ | 9/10 | ND |
| >2000 mm$^3$ | 0/6[b] | ND |
| MC-C <500 mm$^3$ | 12/14 | 6/6 |
| >2000 mm$^3$ | 7/15[c] | 1/5[c] |
| M3 <500 mm$^3$ | 6/6 | 6/6 |
| >2000 mm$^3$ | 3/8[d] | 2/6[d] |

[a]primary tumour volume at the day of the secondary implant. [b]$P<0.001$ ($\chi^2$ test). [c]$P<0.01$ ($\chi^2$ test). [d]$P<0.05$ ($\chi^2$ test). ND, not determined.

LB or MC-B tumour. As shown in Table II, concomitant resistance against a second implant with a different tumour could only be observed when the primary tumour was large at the time of the second implant. These observations suggest that the first peak of concomitant resistance induced by strongly immunogenic tumours (see Figure 1) was specific but the second peak, shared by large tumours independently of their immunogenicity, had some degree of non-specificity.

### *Humoral and cellular immunity in mice bearing immunogenic tumours*

*Humoral immunity* Serum from BALB/c mice bearing the highly immunogenic L15 or MC-C tumour was studied at different stages of tumour development, looking for cytotoxic antibodies anti-L15 or anti-MC-C. The titre of cytotoxic antibodies ($CU_{50}$ $ml^{-1}$) was determined using a $^{51}Cr$ release assay. As shown in Figure 2, cytotoxic antibodies against L15 or MC-C cells were detected only when the tumour was small. Concordant results were obtained with an immunofluorescence assay (data not shown).

*Cellular immunity* As shown in Figure 2, spleen cells from euthymic mice bearing small L15 or MC-C tumours exhibited significant cytotoxicity against $^{51}Cr$-labelled tumour cells. Afterwards, when tumour size increased, this cytotoxic activity soon disappeared in mice bearing MC-C tumour whereas it slowly decayed but did not disappear, in mice bearing L15 tumour. No *in vitro* anti-tumour cytotoxic activity was ever detected when spleen cells from tumour-bearing nude mice were used (data not shown).

Similar results were obtained when anti-tumour immunity was tested using two *in vivo* assays: the adoptive transference and the Winn test. In effect, as shown in Table III, significant resistance to the development of s.c. $5 \times 10^5$ MC-C tumour cells could be adoptively transferred by a single i.v. injection of $10^8$ spleen cells from mice bearing small MC-C tumours but not from mice bearing large tumours. Similarly, when $50 \times 10^6$ spleen cells from mice bearing MC-C tumours were assayed in a Winn test against MC-C tumour cells, only spleen cells from mice bearing small tumours showed some detectable anti-tumour activity.

In order to test natural killer (NK) activity, spleen cells from MC-C-bearing mice were *in vitro* assayed against $^{51}Cr$-labelled YAC-1 cells. In tumour-bearing euthymic mice, NK activity was lower than normal at every stage of tumour growth. In tumour-bearing nude mice, NK values higher than normal were seen when MC-C was small; when MC-C was larger NK activity gradually decreased to normal values (data not shown).

*Inhibitory activity in serum of tumour-bearing hosts against tumour cells* in vitro

Serum collected from tumour-bearing BALB/c mice exhibited, in a [$^3$H]thymidine uptake assay, an inhibitory activity on *in vitro* proliferation of the same tumour cells. Serum was routinely decomplemented before use (when non-decomplemented serum was used, similar results were obtained). As can be seen in Figure 3, this activity was proportional to tumour size ($P<0.05$, Spearman correlation); individual values taken from one experiment chosen as example, are shown in Table IV. This inhibitory activity correlated with the intensity of the second peak of concomitant resistance ($P<0.02$, Spearman correlation); that is, the higher the second peak of concomitant resistance, the stronger the inhibitory activity found in the serum of tumour-bearing mice; for example, LB-bearing mice exhibited the highest second peak of concomitant resistance (see Figure 1) and

**Figure 2** Cytotoxic activity in serum or spleen cells from BALB/c mice bearing L15 (open symbols) or MC-C (filled symbols) tumours, against $^{51}$Cr-labelled tumour cells. Complement-dependent cytotoxic antibodies: each point represents the mean±s.e. of two experiments for L15 (○–○) or MC-C (●–●) tumours. $P<0.01$ for small L15 tumour; $P<0.05$ for small MC-C tumour (*t*-test). Cytotoxic activity of spleen cells: each point represents the mean±s.e. of four determinations for L15 (□–□) or MC-C (■–■) tumours. (- - -: spleen cells from BALB/c mice immunised against L15). $P<0.01$ for small MC-C and L15 tumours; $P<0.05$ for larger L15 tumours (*t*-test).

**Table III** Adoptive transference of splenocytes and Winn test against MC-C tumour

| *Origin of spleen cells* | *Survival index[a] Assay* *Adoptive transference* | *Winn test* |
|---|---|---|
| Normal mice | 42.1±5.9 ($n$=6) | 53.5±2.6 ($n$=8) |
| Mice bearing MC-C tumour of 200–500 mm$^3$ | 65.5±7.9[b] ($n$=11) | 110.8±16.1[c] ($n$=7) |
| Mice bearing MC-C tumour of 700–900 mm$^3$ | 78.6±6.5[c] ($n$=10) | 124.2±18.1[c] ($n$=7) |
| Mice bearing MC-C tumour of 2700–2900 mm$^3$ | 51.6±4.1 ($n$=3) | 50.5±1.5 ($n$=2) |

In the adoptive transference assay, normal BALBc/mice were i.p. inoculated with $10^8$ spleen cells from normal or MC-C bearing mice and, 2 h later, they received a s.c. challenge with $5\times10^5$ MC-C cells. In the Winn test, $50\times10^6$ spleen cells and $5\times10^5$ MC-C cells were s.c. innoculated together. [a]Survival index: survival time in days/($t/n$)±s.e. where $t$ is the number of mice that died of tumour and $n$ is the total number of mice inoculated. [b]$P<0.05$ (*t*-test). [c]$P<0.001$ (*t*-test).

**Figure 3** Growth-inhibitory activity of serum from tumour-bearing euthymic and nude BALB/c mice, measured by a [$^3$H]thymidine uptake assay and expressed as the ratio between GIU50 ml$^{-1}$ of serum from tumour-bearing euthymic (●–●) or nude (○–○) mice and GIU50 ml$^{-1}$ of normal serum (- - - -). Each point represents the mean±s.e. of 2–12 experiments. Number of tumour-bearing mice used ranged from 20 to 124 per tumour. The titre of inhibitory activity reached the highest values when tumour became large: L15: $P<0.01$, MC-D: $P<0.05$, MC-C: $P<0.05$, MNUMPA-1.1: $P<0.05$, MC-B: $P<0.01$, MNU: $P<0.05$, M3: $P<0.01$ and LB: $P<0.01$ (*t*-test).

**Table IV** Effect of serum from BALB/c mice bearing MC-C tumour, on *in vitro* MC-C proliferation

| *Final serum dilution* | *[³H]thymidine uptake by MC-C cells (c.p.m.±s.d.)*[a] Group 1[b] | Group 2[c] | Group 3[d] | Normal serum |
|---|---|---|---|---|
| 1:2 | 18 244±9126 | 17 235±2405 | 3734±497 | 47 713±3435 |
| 1:4 | 65 804±9196 | 46 892±3539 | 20 619±4877 | 68 134±1315 |
| 1:8 | 83 286±3576 | 60 916±6327 | 36 193±4396 | 79 042±8056 |
| 1:16 | 91 148±7699 | 74 732±7744 | 54 184±3700 | 88 787±3504 |
| $GIU_{50}$ $ml^{-1}$ | 31.5 | 43 | 126.7 | 20.5 |
| Ratio | 1.5 | 2.1 | 6.2 | 1 |

[a]Each value is the mean of triplicate to sextuplicate measurements; c.p.m. incorporated by MC-C in medium only = 95664 ± 3834. [b]Serum from mice bearing an MC-C tumour of 200–300 $mm^3$. [c]Serum from mice bearing an MC-C tumour of 1500–1800 $mm^3$. [d]Serum from mice bearing an MC-C tumour of 2700–2900 $mm^3$

**Figure 4** Decay of serum growth-inhibitory activity and abrogation of resistance to a 'second' tumour challenge in MC-C tumour-excised mice. Twelve MC-C tumour-bearing mice (1800–2000 $mm^3$) were divided into two groups: in six mice, tumour was excised whereas in the remaining six mice, tumour remained undisturbed. (**a**) Serum growth-inhibitory activity was measured when tumour was present (day 0) or 5, 10 and 17 days after tumour excision. Ordinates: ratio of $GIU_{50}$ of serum from tumour-excised mice to $GIU_{50}$ of normal serum. Dashed line: normal serum. Each bar represents the mean ± s.e. of six sera. Day 0: $P<0.01$ and day 5: $P<0.05$ as compared with normal serum; days 10 and 17, $P$: NS. (**b**) Seven days after tumour excision (indicated with the arrow), excised ($n=6$), tumour-bearing ($n=6$) and control ($n=7$) mice received an implant of $1\times10^5$ MC-C tumour cells in the opposite flank and tumour growth of this implant was evaluated. ■–■: tumour-excised. △–△: tumour-bearing. ○–○: control mice. Differences between tumour-excised and tumour-bearing: $P<0.01$ at days 12, 15 and 20. Differences between tumour-excised and control were non significant at every day tested.

correlatively their sera exhibited the strongest inhibitory activity. Reciprocally, C7HI-bearing mice did not show the second peak of concomitant resistance and correlatively their sera did not exhibit any inhibitory activity. The remaining tumours exhibited intermediate values in both the second peak of concomitant resistance and the inhibitory activity in serum. The high titre of *in vitro* growth-inhibitory activity, as evaluated by the [³H]thymidine uptake assay, found in sera from mice bearing large tumours, (Figure 3) strongly contrasts with the absence of cytotoxic antibodies as evaluated by the $^{51}Cr$ release assay in the same sera (Figure 2). Furthermore, sera from mice that had been immunised against L15 (but not bearing the tumour) exhibiting a high titre of cytotoxic antibodies (Figure 2), did not show any inhibitory activity on *in vitro* tumour proliferation (data not shown).

Similar results to those obtained in euthymic mice were registered in nude mice (Figure 3), suggesting that this inhibitory activity is thymus independent.

*Dependence on primary tumour* Serum inhibitory activity from mice bearing a large LB (>2000 $mm^3$) tumour was dependent on the presence of a growing tumour; in effect, 48 h after tumour excision, serum inhibitory activity dropped from 223.2 ± 59.2 $GIU_{50}$ $ml^{-1}$ (mean of three experiments, ratio to normal serum: 7) to normal values (32.1 ± 5.6 $GIU_{50}$ $ml^{-1}$). Similar observations were carried out in mice bearing a large MC-C tumour (>2000 $mm^3$): after tumour removal, titre of serum inhibitory activity decayed progressively and, correlatively, resistance against a 'secondary' MC-C tumour challenge was abrogated. In effect, while in tumour-bearing mice the second tumour challenge was significantly inhibited, in tumour-excised and normal mice it grew rapidly (Figure 4).

*Transference of growth inhibitory activity* Attempts to transfer resistance against the MC-C tumour by serial i.v. and i.p. inoculation of serum from mice bearing a large MC-C tumour to normal mice, have been unsuccessful. However, transference of resistance was carried out through cross-circulation using parabiotic mice: four mice bearing an MC-C tumour were joined in parabiosis with four normal mice. Seven days later, when tumour volume was >2000 $mm^3$, normal partners of each pair received a challenge of $1\times10^5$ MC-C tumour cells and the growth of this challenge was evaluated; four normal mice (joined in parabiosis with a normal partner) similarly challenged were used as control. Fifteen days after challenge, tumour volume was 157.5 ± 19.6 $mm^3$ in normal partners of (tumour bearing–normal) pairs, while it was 445.3 ± 91.9 $mm^3$ in control mice ($P<0.02$; $t$-test). This mimicry of concomitant resistance correlated with the appearance of serum growth- inhibitory activity in the normal partner (83.4 ± 13.6 $GIU_{50}$ $ml^{-1}$; ratio to normal serum: 2.2; $P<0.05$), transferred from the MC-C-bearing partner (122.1 ± 37.9 $GIU_{50}$ $ml^{-1}$; ratio to normal: 3.2). Titre in normal mice was 38.5 ± 1.4 $GIU_{50}$ $ml^{-1}$.

*Non-specificity* Serum from mice bearing an MC-C tumour >2000 $mm^3$ inhibited the *in vitro* proliferation not only of the proper MC-C but also of the unrelated LB tumour cells using the [³H]thymidine uptake assay. Titre of MC-C serum against MC-C (mean±s.e. of three experiments) was 202.9±12.1 $GIU_{50}$ $ml^{-1}$ (ratio to normal serum: 4.4; $P<0.01$) and against LB (mean±s.e. of three experiments) was 161.7±33.7 $GIU_{50}$ $ml^{-1}$ (ratio to normal serum: 3.1; $P<0.05$). The non-specific *in vitro* inhibition of LB tumour cell proliferation by serum from mice bearing a large MC-C tumour paralleled the non-specific *in vivo* inhibition of a second implant of LB in mice bearing a large MC-C tumour (see Table II).

*Physical properties and serum fractionation* Serum from mice bearing an MC-C tumour >2000 $mm^3$ was subjected to dialysis (12 500 molecular weight cut-off). The inhibitory activity, as evaluated by the [$^3$H]thymidine uptake assay, was only detected in the dialysable fraction, indicating a molecular weight below 12 500. Additionally, this serum activity proved to be resistant to heating at 56°C for 30 min and at 100°C for 5 min, and it remained stable when samples were stored for 2 days at 4°C. Similar properties were exhibited by the growth-inhibitory activity found in serum from mice bearing the non-immunogenic LB tumour (Ruggiero *et al*., 1990). Partial purification of this inhibitory activity was initiated: the active dialysable fraction of serum was concentrated by lyophilisation and applied to a column of Sephadex G-15, and one peak of inhibitory activity was eluted at fractions corresponding to a molecular weight of approximately 1000. This peak was lyophilised and further purified with an HPLC column; 23 peaks were obtained but growth-inhibitory activity (143.5±13.5 $GIU_{50}$ $ml^{-1}$, ratio to normal values: 7.2; $n=3$ experiments; $P<0.001$) was recovered in only one (peak number 8), which presented maximum absorption at 215 and 266 nm (Figure 5).

**Figure 5** HPLC elution profile of the growth-inhibitory activity found in the serum from LB tumour-bearing mice. The growth-inhibitory activity of each fraction was assayed on LB tumour cells using the [$^3$H]thymidine uptake assay. Arrow indicates the only fraction (number 8) with inhibitory activity. Absorbance was registered at 215 (**a**) [(- - - -), acetonitrile] and 266 (**b**) nm. Inset: Absorption pattern of fraction number 8.

### Discussion

Concomitant resistance has been described in mice bearing both immunogenic and non-immunogenic tumours, but to date, the mechanisms involved had been reported as being very different. In this paper, using nine murine tumours with different degrees of immunogenicity, we have tested whether, besides those differences, a common mechanism could underlie the concomitant resistance induced by all tumours, independently of their immunogenicity.

Our results suggest that, during the primary tumour development, two temporally separate peaks of concomitant resistance can be detected. The first peak was observed when the primary tumour was small; it was tumour specific and thymus dependent as it was present in euthymic but not in nude mice; its intensity was proportional to tumour immunogenicity and a typical immunological rejection was observed histologically at the site of the second tumour implant undergoing concomitant resistance. Furthermore, the kinetics of appearance and disappearance of the first peak of concomitant resistance paralleled the kinetics of appearance and disappearance of cytotoxic antibodies and cell-mediated cytotoxicity against the tumour; NK cells did not seem to play a main role. On the other hand, the second peak of concomitant resistance was induced by both immunogenic and non-immunogenic large tumours; it was non specific and thymus independent as it was exhibited in both euthymic and nude mice and it did not correlate with tumour immunogenicity; neither cytotoxic antibodies nor cellular immune responses were involved. No host cell infiltration but morphologically well-preserved tumour cells were histologically detected at the site of the second tumour implant undergoing concomitant resistance, suggesting a cytostatic mechanism. The intensity of the second peak of concomitant resistance correlated with the activity of serum factors (different from antibodies or complement) that inhibited the *in vitro* proliferation of tumour cells; that is, the higher the second peak, the stronger the inhibitory activity. Reciprocally, when this serum inhibitory activity was absent (the only case was the highly metastatic C7HI tumour) the second peak did not appear.

The relationship between the second peak of concomitant resistance and serum inhibitory factors is additionally supported by the following evidence: (a) the *in vivo* non-specific resistance to a second tumour challenge paralleled the *in vitro* non-specific antiproliferative activity by serum from mice bearing large tumours; (b) removal of a large tumour–immunogenic or non-immunogenic–was accompanied by a decay in the inhibitory activity in serum simultaneously with the disappearance of resistance to a second tumour challenge; (c) transference of both serum growth-inhibitory activity and resistance to a tumour challenge from immunogenic or non-immunogenic tumour-bearing to normal mice was achieved by cross-circulation using parabiotic mice.

Serum inhibitory factors from mice bearing both immunogenic and non-immunogenic tumours proved to be heat resistant and dialysable; further purification showed that this inhibitory activity was eluted from a Sephadex G-15 column at fractions corresponding to a molecular weight of approximately 1000 and it was recovered from an HPLC column in only one peak presenting maximum absorption at 215 and 266 nm. Although final purification and characterisation of this factor(s) is the subject of a forthcoming paper, data presented here suggest that previously characterised growth inhibitors such as interferons, tumour necrosis factors, the transforming growth factor beta family (Trincheri and Perussia, 1985; Beutler and Cerami, 1986; Keski-Oja *et al*., 1988) and the novel angiostatin (O'Reilly *et al*., 1994) would not be involved because of their larger molecular weight and other physical properties (e.g. tumour necrosis factors, the transforming growth factor beta family and angiostatin do not resist boiling). The source of this inhibitory activity remains speculative; up to now it has been only occasionally recovered in conditioned medium of MC-C

and LB cultures; on the other hand, thymectomy or splenectomy did not alter the titre of serum growth-inhibitory activity (unpublished results). The fact that the serum inhibitory activity disappears after tumour excision could suggest that the tumour cells are responsible for its elaboration; however, an indirect effect of the tumour on the microenvironment can not be discarded.

Previous studies using non-immunogenic tumours have described the existence of concomitant resistance at late stages of tumour growth (here referred to as second peak of concomitant resistance) and a serum-mediated mechanism was suggested (Ruggiero *et al.*, 1990; Prehn, 1993). On the contrary, most of the authors studying concomitant resistance induced by strongly immunogenic tumours have described the existence of the first peak but not that of the second peak of concomitant resistance (Belehradek *et al.*, 1972; Chandradasa, 1973; Vaage, 1973; Howell *et al.*, 1975; Berendt *et al.*, 1978; Leveson *et al.*, 1979; Finlay-Jones *et al.*, 1980; Tuttle *et al.*, 1983). There are three possible explanations for this. First, many of these studies were restricted to relatively early stages of tumour development (Belehradek *et al.*, 1972; Chandradasa, 1973; Vaage, 1973; Howell *et al.*, 1975; Berendt *et al.*, 1978; Leveson *et al.*, 1979; Finlay-Jones *et al.*, 1980). Second, the evaluation of concomitant resistance was often not completely adequate; in effect, most of those studies were carried out using only one dose of tumour cells as the second challenge (Vaage, 1973; Howell *et al.*, 1975; Berendt *et al.*, 1978; Leveson *et al.*, 1979; Finlay-Jones, 1980). Third, in some experiments, concomitant resistance could not be strictly tested as the primary tumour was excised soon after the second challenge (Vaage, 1973, 1977). Our results, describing two temporally separate peaks of concomitant resistance, may explain apparently contradictory results reported by different authors: for example, both Berendt *et al.* (1978) and Gorelik (1983) have worked with the same tumour, the strongly immunogenic Meth A, using similar tumour doses for the first and the second challenges; however, whereas Berendt *et al.* stated that resistance to a second Meth A tumour challenge was observed transiently in mice bearing a very small Meth A tumour, Gorelik stated that resistance to a second Meth A tumour challenge did not appear when the primary tumour was small while its intensity was progressively higher as the primary tumour became larger. In our opinion, the reason for these seemingly contradictory conclusions resides in the different stages of primary tumour growth at which each of these authors has looked for concomitant resistance. Other examples of apparently contradictory but in fact, in our opinion, complementary results, are found in papers studying concomitant resistance associated with melanoma B16 (Leveson, 1979; Gorelik *et al.*, 1981), murine lung Lewis carcinoma 3LL (De Wys, 1972; Gorelik, 1981), rat carcinoma of Flexner-Jobling (Woglom, 1929), methylcholanthrene-induced fibrosarcomas (Deckers *et al.*, 1973; Kearney and Nelson, 1973; Nomi *et al.*, 1986).

In conclusion, the data presented in this paper suggest for the first time, to our knowledge, that besides the classical immunological mechanism of concomitant resistance induced by small immunogenic tumours, a common mechanism, mediated by serum factors of low molecular weight, seems to underlie the concomitant resistance induced by both immunogenic and non-immunogenic tumours at late stages of primary tumour development.

**Acknowledgements**
We are grateful to Drs MI Piazzon, MA Isturiz, SL Rabasa and RT Prehn for critical discussion of this manuscript and to Drs C Lanari, I Nepomnaschy, G Dran, S Torello and RP Meiss for offering many helpful suggestions. The authors wish to thank JJ Portaluppi and A Morales especially for excellent technical assistance.

This work was supported by CONIČET (Consejo Nacional de Investigaciones Científicas y Técnicas) and FUNDALEU (Fundación para combatir la leucemia). M Franco, PD di Gianni and A Goldman are fellows of CONICET. D Bustuoabad, CD Pasqualini and RA Ruggiero are members of Research Career, CONICET.

### References

AKPORIAYE ET, KUDALORE M, STEVENSON AP, KRAEMER PM AND STEWART CC. (1988). Isolation and reactivity of host effectors associated with the manifestation of concomitant tumor immunity. *Cancer Res.*, **48,** 1153–1158.

ATTIA MA AND WEISS DW. (1966). Immunology of spontaneous mammary carcinomas in mice. V. Acquired tumor resistance and enhancement in strain A mice infected with mammary tumor virus. *Cancer Res.*, **26,** 1787–1800.

BELEHRADEK J, BARSKI G AND THONIER M. (1972). Evolution of cell-mediated antitumor immunity in mice bearing a syngeneic chemically induced tumor. Influence of tumor growth, surgical removal and treatment with irradiated tumor cells. *Int. J. Cancer*, **9,** 461–469.

BERENDT MJ, NORTH RJ AND KIRSTEIN DP. (1978). The immunological basis of endotoxin-induced tumor regression. *J. Exp. Med.*, **148,** 1560–1569.

BEUTLER B AND CERAMI A. (1986). Cachectin and tumour necrosis factor as two sides of the same biological coin. *Nature*, **320,** 584–588.

BONFIL RD, RUGGIERO RA, BUSTUOABAD OD, MEISS RP AND PASQUALINI CD. (1988). Role of concomitant resistance in the development of murine lung metastases. *Int. J. Cancer*, **41,** 415–422.

BONFIL RD, SORASIO MC, LUCERO GRITTI MF, BUSTUOABAD OD, MEISS RP, KORDON E, LANARI C AND PASQUALINI CD. (1989). Caracterización del adenocarcinoma mamario murino C7HI, un nuevo modelo para el estudio de las metástasis. *Medicina* (Buenos Aires) **49,** 479.

BRUNNER KT, MAUEL J, CEROTTINI JC AND CHAPUIS B. (1968). Quantitative assay of the lytic action of immune lymphoid cells on $^{51}$Cr-labelled allogeneic target cells *in vitro*; inhibition by isoantibody and by drugs. *Immunology*, **14,** 181–196.

CHANDRADASA KD. (1973). The development and specific suppression of concomitant immunity in two syngeneic tumour-host systems. *Int. J. Cancer*, **11,** 648–662.

DECKERS PJ, DAVIS RC, PARKER GA AND MANNICK JA. (1973). The effect of tumor size on concomitant tumor immunity. *Cancer Res.*, **33,** 33–39.

DE WYS WD. (1972). Studies correlating the growth rate of a tumor and its metastases and providing evidence for tumor-related systemic growth-retarding factors. *Cancer Res.*, **32,** 374–379.

FINLAY-JONES JJ, BARTHOLOMAEUS WN, FIMMEL PJ, KEAST D AND STANLEY NF. (1980). Biologic and immunologic studies on a murine model of regional lymph node metastasis. *J. Natl Cancer Inst.* **64,** 1363–1369.

GORELIK E. (1983). Resistance of tumor-bearing mice to a second tumor challenge. *Cancer Res.*, **43,** 138–145.

GORELIK E, SEGAL S AND FELDMAN M. (1981). On the mechanism of tumor 'concomitant immunity'. *Int. J. Cancer*, **27,** 847–856.

HOWELL SB, DEAN JH AND LAW LW. (1975). Defects in cell-mediated immunity during growth of a syngeneic simian virus-induced tumor. *Int. J. Cancer*, **15,** 152–169.

KEARNEY R AND NELSON DS. (1973). Concomitant immunity to syngeneic methylcholanthrene-induced tumours in mice. Occurrence and specificity of concomitant immunity. *Aust. J. Exp. Biol. Med. Sci.*, **51,** 723–735.

KESKI-OJA J, POSTLETHWAITE AE AND MOSES HL. (1988). Transforming growth factors in the regulation of malignant cell growth and invasion. *Cancer Invest.*, **6,** 704–724.

KLEIN S, COLOMBO LL, STILLITANI-D'ELÍA I AND BONAPARTE YP. (1980). Diferente inmunogenicidad de dos tumores de mama murinos con distinta capacidad metastásica en pulmón. *Medicina* (Buenos Aires), **40,** 826–827.

KORDON E, LANARI C, MOLINOLO AA, ELIZALDE PV, CHARREAU EH AND PASQUALINI CD. (1991). Estrogen inhibitor of MPA-induced mouse mammary tumor transplants. *Int. J. Cancer*, **49**, 900–905.

LEVESON SH, HOWELL JH, PAOLINI NS, TAN MH, HOLYOKE ED AND GOLDROSEN MH. (1979). Correlations between the leukocyte adherence inhibition microassay and in vivo tests of transplantation resistance. *Cancer Res.*, **39**, 582–586.

LOWRY OH, ROSEBROUGH HF, FARR ML AND RANDALL RF. (1951). Protein measurement with the Folin phenol reagent. *J. Biol. Chem.*, **143**, 265–275.

MEISS RP, BONFIL RD, RUGGIERO RA AND PASQUALINI CD. (1986). Histologic aspects of concomitant resistance induced by non-immunogenic murine tumors. *J. Natl Cancer Inst.*, **76**, 1163–1175.

NOMI S, NAITO K, KAHAN BD AND PELLIS NR. (1986). Effects of concomitant and sinecomitant immunity on postsurgical metastasis in mice. *Cancer Res.*, **46**, 6111–6115.

NORTH RJ. (1984). The murine antitumor immune response and its therapeutic manipulation. *Adv. Immunology*, **35**, 89–155.

O'REILLY MS, HOLMGREN L, SHING Y, CHEN C, ROSENTHAL RA, MOSES M, LANE WS, CAO Y, SAGE EH AND FOLKMAN J. (1994). Angiostatin: A novel angiogenesis inhibitor that mediates the suppression of metastases by a Lewis lung carcinoma. *Cell*, **79**, 315–328.

PASQUALINI CD AND COLMERAUER MEM. (1976). Immunological enhancement of a murine allogeneic lymphoma. *Medicina* (Buenos Aires), **36**, 189–192.

PAZOS P, LANARI C, MEISS RP, CHARREAU EH AND PASQUALINI CD. (1992). Mammary carcinogenesis induced by *N*-methyl-*N*-nitrosourea (MNU) and medroxyprogesterone acetate (MPA) in BALB/c mice. *Breast Cancer Res. Treatment*, **20**, 133–138.

PREHN RT. (1993). Two competing influences that may explain concomitant tumor resistance. *Cancer Res.*, **53**, 3266–3269.

RAO VS AND BONAVIDA B. (1976). Specific enhancement of tumor growth and depression of cell-mediated immunity following sensitization to soluble tumor antigens. *Cancer Res.*, **36**, 1384–1391.

RAO VS, BONAVIDA B, ZIGHELBOIM J AND FAHEY JL. (1974). Preferential induction of serum blocking activity and enhancement of skin allograft by soluble alloantigen. *Transplantation*, **17**, 568–575.

RUGGIERO RA, BUSTUOABAD OD, BONFIL RD, MEISS RP AND PASQUALINI CD. (1985). 'Concomitant immunity' in murine tumours of non-detectable immunogenicity. *Br. J. Cancer*, **51**, 37–48.

RUGGIERO RA, BUSTUOABAD OD, CRAMER P, BONFIL RD & PASQUALINI CD. (1990). Correlation between seric antitumor activity and concomitant resistance in mice bearing non-immunogenic tumors. *Cancer Res.*, **50**, 7159–7165.

TRINCHERI G AND PERUSSIA B. (1985). Immune interferon: a pleiotropic lymphokine with multiple effects. Immunol. *Today*, **6**, 131–136.

TUTTLE RL, KNICK VC, STOPFORD CR AND WOLBERG G. (1983). In vivo and in vitro antitumor activity expressed by cells of concomitantly immune mice. *Cancer Res.*, **43**, 2600–2605.

VAAGE J. (1973). Influence of tumor antigen on maintenance versus depression of tumor-specific immunity. *Cancer Res.*, **33**, 493–503.

VAAGE J. (1977). Host serum factors versus tumor factors in immune resistance to metastases. In *Cancer Invasion and Metastasis. Biologic Mechanisms and Therapy*, Day SB Myers W, Stansly P, Garattini S AND Lewis M (eds) pp. 305–318. Raven Press: New York.

WINN HJ. (1961). Immune mechanisms in homotransplantation II. Quantitative assay of the immunological activity of lymphoid cells stimulated by tumour homograft. *J. Immunol.*, **86**, 228–239.

WOGLOM WH. (1929). Immunity to transplantable tumours. *Cancer Rev.*, **4**, 129–209.

ZAHALKA MA, OKON E AND NAOR D. (1993). Blocking lymphoma invasiveness with a monoclonal antibody directed against the beta-chain of the leukocyte adhesion molecule (CD18). *J. Immunol.*, **150**, 4466–4477.